# The reproducibility of psychiatric evaluations of work disability: two reliability and agreement studies

**DOI:** 10.1186/s12888-019-2171-y

**Published:** 2019-07-03

**Authors:** Regina Kunz, David Y. von Allmen, Renato Marelli, Ulrike Hoffmann-Richter, Joerg Jeger, Ralph Mager, Etienne Colomb, Heinz J. Schaad, Monica Bachmann, Nicole Vogel, Jason W. Busse, Martin Eichhorn, Oskar Bänziger, Thomas Zumbrunn, Wout E. L. de Boer, Katrin Fischer

**Affiliations:** 1 Department of Clinical Research, Evidence-based Insurance Medicine, University of Basel, University Hospital, 4031 Basel, Switzerland; 2 Swiss Society of Insurance Psychiatry, SGVP, 4051 Basel, Switzerland; 3 Private Practice for Psychiatry, 4051 Basel, Switzerland; 4 Swiss National Accident Insurance Funds, 6004 Luzern, Switzerland; 5 Private Practice for Psychiatry and Psychotherapy, 6004 Lucerne, Switzerland; 6 Institute of Medical Disability Evaluations of Central Switzerland, 6003 Lucerne, Switzerland; 7 Psychiatric University Hospital Basel, 4002 Basel, Switzerland; 8 French-Speaking Swiss Association of Practitioners in Medical Expertise (ARPEM), 1025 St Sulpice, Switzerland; 9 Institute for Medical Disability Evaluation Interlaken, 3800 Unterseen, Switzerland; 10 Department of Anaesthesia, McMaster University, Hamilton, L8S 4K1 ON Canada; 11 Department of Health Research Methods, Evidence and Impact, McMaster University Hamilton, Hamilton, L8S 4K1 ON Canada; 12 Private Practice for Psychiatry, 4057 Basel, Switzerland; 13 Zuerich Office of the Swiss National Disability Insurance, 8005 Zürich, Switzerland; 14 Institute Humans in Complex Systems, School of Applied Psychology, University of Applied Sciences Northwestern Switzerland, 4600 Olten, Switzerland

**Keywords:** Disability evaluation, Work capacity evaluation, Return to work, Social security, Reproducibility of results, Observer variation, Evidence-based medicine

## Abstract

**Background:**

Expert psychiatrists conducting work disability evaluations often disagree on work capacity (WC) when assessing the same patient. More structured and standardised evaluations focusing on function could improve agreement. The RELY studies aimed to establish the inter-rater reproducibility (reliability and agreement) of ‘functional evaluations’ in patients with mental disorders applying for disability benefits and to compare the effect of limited versus intensive expert training on reproducibility.

**Methods:**

We performed two multi-centre reproducibility studies on standardised functional WC evaluation (RELY 1 and 2). Trained psychiatrists interviewed 30 and 40 patients respectively and determined WC using the Instrument for Functional Assessment in Psychiatry (IFAP). Three psychiatrists per patient estimated WC from videotaped evaluations. We analysed reliability (intraclass correlation coefficients [ICC]) and agreement (‘standard error of measurement’ [SEM] and proportions of comparisons within prespecified limits) between expert evaluations of WC. Our primary outcome was WC in alternative work (WC_alternative.work_), 100–0%. Secondary outcomes were WC in last job (WC_last.job_), 100–0%; patients’ perceived fairness of the evaluation, 10–0, higher is better; usefulness to psychiatrists.

**Results:**

Inter-rater reliability for WC_alternative.work_ was fair in RELY 1 (ICC 0.43; 95%CI 0.22–0.60) and RELY 2 (ICC 0.44; 0.25–0.59). Agreement was low in both studies, the ‘standard error of measurement’ for WC_alternative.work_ was 24.6 percentage points (20.9–28.4) and 19.4 (16.9–22.0) respectively. Using a ‘maximum acceptable difference’ of 25 percentage points WC_alternative.work_ between two experts, 61.6% of comparisons in RELY 1, and 73.6% of comparisons in RELY 2 fell within these limits. Post-hoc secondary analysis for RELY 2 versus RELY 1 showed a significant change in SEM_alternative.work_ (− 5.2 percentage points WC_alternative.work_ [95%CI − 9.7 to − 0.6]), and in the proportions on the differences ≤ 25 percentage points WC_alternative.work_ between two experts (*p* = 0.008). Patients perceived the functional evaluation as fair (RELY 1: mean 8.0; RELY 2: 9.4), psychiatrists as useful.

**Conclusions:**

Evidence from non-randomised studies suggests that intensive training in functional evaluation may increase agreement on WC between experts, but fell short to reach stakeholders’ expectations. It did not alter reliability. Isolated efforts in training psychiatrists may not suffice to reach the expected level of agreement. A societal discussion about achievable goals and readiness to consider procedural changes in WC evaluations may deserve considerations.

**Electronic supplementary material:**

The online version of this article (10.1186/s12888-019-2171-y) contains supplementary material, which is available to authorized users.

## Background

Western countries have social security systems in place that provide wage replacement benefits to individuals whose reduced health restricts or prevents them from working [1]. Over the last decade, most countries of the Organisation for Economic Co-operation and Development (OECD) have reported escalating rates of disabled workers, with current estimates ranging between four to eight individuals per thousand of working age population per year [2, 3]. In absolute terms, the annual number of new recipients of disability benefits ranges between 16,000 individuals for Switzerland and 1,700,000 for the USA. These numbers constitute a substantial economic challenge for society.

Many treating psychiatrists [3, 4] are engaged to perform medical evaluations, aimed at clarifying functional capacity of workers who claim inability to work due to illness or injury. Work capacity (WC) evaluations integrate detailed information about patients’ jobs, their functioning at work, residual ability to perform job-specific skills, and self-perceived work ability. This process involves a number of implicit and explicit judgements. The experts’ final judgement is further influenced by their interaction with patients, personal experiences, training, personal and societal norms and values [5]. This complexity calls for a rigorously structured approach to medical evaluations, with clear guidance on the process for acquiring and integrating information.

Our research on the reproducibility of WC evaluations evolved from widespread dissatisfaction with medical evaluations in Switzerland, where two nationwide surveys highlighted serious concerns regarding psychiatric evaluations of WC [3, 6]. Respondents ranked the missing link between expert findings and their final judgement on work incapacity as their top concern. Moreover, a systematic review on work disability evaluations from 12 countries revealed low reproducibility [7]. Almost all countries lacked an evidence-based approach to address the complexity of the task [8, 9]. We developed and piloted a functional evaluation programme that was intended to close the gap between health complaints and work limitations, and thereby increase transparency and uniformity of WC evaluations [10, 11].

Reproducibility is an umbrella term that encompasses two related concepts [12, 13]. First, the reliability of a ‘measuring device’, which –in our context– means how well expert judgements can distinguish patients with different degrees of WC from each other, despite measurement errors. Second agreement, which assesses how close the scores for repeated measurements (by the same or different raters) are for the same individual, and therefore concerns measurement error.

Good reproducibility, which encompasses both reliability and agreement, is a prerequisite for implementing a procedure in routine practice. We explored the effect of standardised training in functional evaluation for psychiatrists assessing the WC of patients reporting disability due to mental illness. We focused on patients with mental disorders, as this population is perceived as being particularly vulnerable to subjectivity regarding the evaluation of work disability [2, 14, 15].

## Methods

Two major administrative governmental changes^1^ interfered with our original research plan - a reproducibility study followed by a randomised controlled trial (RCT) on work disability evaluations based on usual practice versus evaluations using functional evaluations [16]. We therefore conducted two reproducibility studies in the same setting, one based on limited training in functional evaluation with delayed application in the study (RELY 1), the second providing intensive standardised and manualised training with timely application [16] (RELY 2).

### Study design and participants

We performed two multi-centre reproducibility studies, RELY 1 and 2, using a partially crossed design in which four expert psychiatrists (one interviewer, three video raters) independently rated the WC of actual patients claiming disability benefits (see study protocol [11], Additional files 1 and 2 for detailed methodology). We followed the Guidelines for Reporting Reliability and Agreement Studies (GRRAS) [13].

In RELY 1, eligible psychiatrists performed disability evaluations commissioned by the National Disability Insurance Scheme or the Swiss National Accident Insurance Fund (Suva). Psychiatrist recruitment took place in five assessment centres. Eligible patients had submitted an application for disability benefits from the Zurich office of the National Disability Insurer or from Suva, were fluent in German, and were attending an independent psychiatric evaluation for the first time. In line with routine procedures of the commissioning organisation^1^, eligible patients were randomly distributed among the assessment centres and allocated to the next available interviewing psychiatrist. Patient recruitment in the five disability assessment centres took place between November 2013 and February 2015.

In RELY 2, all but one RELY 1-experts were recruited as interviewers. The recruitment of new video raters was carried out through Swiss Insurance Medicine, the professional society of insurance medicine. Patient recruitment for RELY 2 followed the procedures of RELY 1 and took place between July 2015 and April 2016. To compensate for the time loss in RELY 1 (see below), RELY 2 re-used 15 videos from RELY 1 that scored highest for functional interviewing criteria [17].

### Procedures

Our functional evaluation approach incorporated three tools to systematically collect and document information for judging the patients’ work disability: (1) a semi-structured interview about their work and self-perceived work limitations; (2) concise descriptions of exemplary reference jobs for alternative work, and (3) a three-part instrument for documenting work-related limitations (ICF-based Instrument for Functional Assessment in Psychiatry, IFAP 1 on mental functions; IFAP 2a&b on functional capacities, based on [18] (with further enhancements in RELY 2), IFAP 3a&b on overall WC, single-item scale from 100 to 0% WC, relating to the patients’ last job [3a] and alternative work [3b]) [10, 11]. IFAP 1 and 2 will be reported elsewhere.

Formal training in RELY 1 included written material, instructions on the use of IFAP [10] and three training sessions with didactic presentations, interactive small group sessions [11] and individual practice between sessions. The governmental changes^1^ and the enforced reorganization in the assessment centres stalled RELY 1 with a mean training-to-rating delay exceeding one year. We named RELY 1 the group ‘with limited training and delayed application’. The rating psychiatrists in RELY 2 underwent an intensive manualised training with expert calibration to the IFAP rating rules and enhanced descriptions of reference jobs, and doubling of training hours followed by timely implementation.

Assigning video raters randomly to patients ensured concealed allocation and prevented rater-group membership where the same raters repeatedly form a rating group for a patient [19]. Video raters reviewed the material independently, unaware of the other raters. Neither patients nor psychiatrists were blinded. Interviewing psychiatrists integrated the functional interview into their usual evaluation which was videotaped. They completed IFAP ratings, and summarized patients’ medical files for the rating psychiatrists. Three psychiatric raters per patient viewed the videos with medical summaries and job descriptions, and completed the IFAP ratings. In total, four independent ratings were generated for each patient.

### Outcomes, data collection, analysis

The primary outcome was expert judgement of patients’ overall WC for alternative work (IFAP 3b) used by the insurers to calculate the patients’ benefits. Secondary outcomes were WC for patients’ last job (IFAP 3a), experts’ certainty in their own judgements of WC (scale 0–10), patients’ perceived fairness of the evaluation (a 29-item questionnaire [20, 21], see Additional file 3), including general satisfaction with the evaluation (scale 0–10), and experts’ perception of the functional evaluation (telephone interviews, RELY 1; online survey, RELY 2).

We collected socio-demographic data on patients, experts, patients’ mental disorder(s) [22] with impact on WC and the experts’ judgement of the disorders’ severity (scale from 0 to 10). To establish patients’ main diagnosis, three of four psychiatrists had to code the same diagnosis on the second digit level of ICD-10 (i.e. F0, F1, etc.). Typicality was ascertained by comparing study patients to patients in usual practice with respect to six predefined criteria [11].

Observations that expert evaluations without standardised procedures typically achieve low reliability (ICC or Kappa around 0.4) [8] informed our sample size calculation. With a sample size of 30 in RELY 1, a two-sided 95%CI around the intraclass correlation coefficient (ICC) would extend + 0.15 from the observed ICC, assuming a true ICC value of 0.6 [23, 24]. The sample size of 40 in RELY 2 accounted for the wider 95%CI observed in RELY 1.

We used descriptive statistics for continuous and categorical data, plotting experts’ ratings of overall WC per patient (‘last job’, ‘alternative work’) and counting patients with maximum divergent WC ratings (i.e., ranging between 100 and 0%) [6]. Variance components (psychiatrists, patients, residuals) underlying the ICC were determined using a linear mixed-effects model. We reported reliability by the ICC variant measuring absolute agreement, ICC_abs.agree_ [25]:

$$ {ICC}_{\mathrm{abs}.\mathrm{agree}}=\frac{\upsigma_{Patients}^2}{\upsigma_{Patients}^2+{\upsigma}_{Psychiatrists}^2+{\upsigma}_{Residuals}^2\ } $$

with  ^*2*^_*Patients*_ (between-patient variance),  ^*2*^_*Psychiatrists*_ (between-psychiatrist variance), and  ^*2*^_*Residuals*_ (residual variance) as a value between 0 and 1. The linear mixed-effects model used WC as response and crossed random intercepts for patients and psychiatrists. An intercept was fitted as the only fixed effect. Model-based parametric bootstrapping was used to estimate 95%CIs. We interpreted the ICC as poor (ICC < 0.40), fair (0.40–0.59), good (0.60–0.74) and excellent (> 0.75) [26].

For agreement, we report 1) standard error of measurement (SEM) and 2) proportion of psychiatrist-by-psychiatrist comparisons that stayed within a prespecified limit for the difference in WC [6, 12]. Agreement parameters retain their actual scale of measurement making clinical interpretations more accessible [13]. ‘Standard error of measurement’ describes the psychiatrist variation in WC [12].

$$ {SEM}_{\mathrm{agreement}}=\sqrt{\upsigma_{Psychiatrists}^2+{\upsigma}_{Residuals}^2} $$

To facilitate the clinical interpretation of the observed ‘standard error of measurement’, we calculated an expected value of ‘standard error of measurement’ [12] based on the results of a recent survey [6] in which more than 600 Swiss stakeholders from five interest groups (psychiatrists, experts, lawyers, judges, insurers) expressed their expectations on what constitutes a ‘maximum acceptable difference’ (Table 1). Expected value of ‘standard error of measurement’ is defined [12] as $SEM\_ expected=\frac{\mathrm{MAD}}{1.96\ast \sqrt{2}}$, where MAD denotes the ‘maximum acceptable difference’ in WC ratings between any two raters (corresponding to the ‘smallest detectable change’ in de Vet 2006 [12]). We used the upper limit of the interquartile range (IQR) of the ‘maximum acceptable difference’ determined by psychiatrists and experts (25 percentage points, see Table 1) and by lawyers, judges and insurers (20 percentage points). We used the upper limit of the IQR rather than the median to indicate that 75% of that stakeholder group considered higher differences in WC ratings as unacceptable. However, many in this group felt that the ‘maximum acceptable difference’ should be as low as 20, 15, or even 10 percentage points.

**Table 1:** Inter-rater variability: Expectation of stakeholders**. ‘**Maximum acceptable difference’ in work capacity (WC) ratings between two experts performing a psychiatric evaluation in the same patient [6]

What is the maximum difference in WC ratings that stakeholders would find acceptable when two experts independently assess the same patient?	Lawyers (*n* = 81)	Psychiatrists (*n* = 242)	Experts (*n* = 114)	Judges (*n* = 47)	Insurers (*n* = 108)
… in the current situation of performing evaluations, median difference (interquartile range, IQR)	15% (10–20%)	20% (10–25%)	20% (10–25%)	15% (10–20%)	10% (10–20%)

Legend: WC: work capacity; % WC = absolute percentage points in work capacity

How to interpret this table?

• 75% of treating and expert psychiatrists felt that the ‘maximum acceptable difference’ in WC ratings between two experts should be 25% corresponding to the upper limit of the IQR

• 75% of lawyers, judges and insurers and 50% of treating and expert psychiatrists felt that the ‘maximum acceptable difference’ in WC ratings between two experts should be 20% WC corresponding to the upper limit of the IQR (jurists) or the median (psychiatrists)

• 50% of lawyers, judges and insurers felt that the ‘maximum acceptable difference’ in WC ratings between two experts should be 15% corresponding to the median

• 25% of all stakeholders felt that the ‘maximum acceptable difference’ in WC ratings between two experts should be 10% corresponding to the lower limit of the IQR

For the stakeholders [6], observed ‘standard error of measurement’ had to be smaller than 9.0 percentage points WC (SEM_expected_ by psychiatrists and experts), and 7.2 percentage points WC (lawyers, judges, insurers). 2) Proportion of comparisons within a prespecified limit: Comparing the ratings of all four psychiatrists per patient with each other resulted in six comparisons per patient. We calculated how often this proportion varied with a threshold of ≤ 10 (15-, 20-, up to 50-) percentage points WC. These thresholds were informed by a Swiss survey [6] with over 600 stakeholders (lawyers, treating psychiatrists, expert psychiatrists, social judges, insurers’ employees) who reported what degree of deviation of assumed WC between two psychiatrists they would find – at maximum – acceptable (Table 1, ‘maximum acceptable difference’, reported as median and interquartile range [IQR]). We used the upper limit of IQR as threshold (i.e., 75% of respondents who approved only equal or lower differences between two raters as acceptable) to determine agreement between study psychiatrists at different levels of stakeholder expectations.

To test whether the psychiatrists systematically differ in their ratings, we formulated two mixed-effects models. The null model consists of percentage WC as the response variable, an intercept as the single fixed effect, and a random intercept for the claimants. The alternative model includes crossed random intercepts for claimants and psychiatrists. A likelihood ratio test was performed to test whether allowing for a separate variance component for the psychiatrists significantly improved model fit. For each test, we reported χ2-statistic and associated *p*-value using Satterthwaite’s approximation of degrees of freedom [27].

### Comparing RELY 1 and RELY 2

RELY 1 and 2 can be conceptualized as two treatment arms of a non-randomised comparative study, with psychiatrists in RELY 1 resembling the control group, having received limited training but probably suffered substantial knowledge decay due to the one-year delay in starting the study. Those in RELY 2 resemble the intervention group with intensive training in functional evaluation and timely application in the study as planned. Both studies had recruited psychiatrists and patients from the same population, patients had received the same procedures and had been rated using the same reporting instrument. We used these similarities to justify post-hoc analyses comparing RELY 1 and 2 [28]. Intensive calibration of experts was expected to decrease psychiatrist variance and total variance, and to reduce maximum divergent ratings among patients.

We used the linear mixed effects model to compare RELY 1 and RELY 2 for difference in percentage WC (‘last job’; ‘alternative work’) and ‘standard error of measurement’ (WC_alternative.work_). We used model-based parametric bootstrapping analogous to estimating the 95%CI of the ICC. Each pair of datasets was compared by fitting the linear mixed-effects models described above and by calculating the differences in percentage WC and ‘standard error of measurement’ (RELY 2 minus RELY 1). The procedure was repeated 9999 times.

### Patient and public involvement

To promote trust in our study, we assembled an observer group of stakeholders from patient organisations, the legal profession (patient lawyers, academics, cantonal courts and the Swiss Federal Supreme Court), professional medical societies and representatives of social security. The group met once a year for update and discussion. Furthermore, we piloted a questionnaire on perceived fairness focusing on comprehension, acceptance, and ease of use with 40 patients from one assessment centre. We communicated study rationale and design online (www.unispital-basel.ch/ebim/RELY).

## Results

### RELY 1-study

Of 160 potentially eligible patients, 109 met inclusion criteria and 30 (28%) entered the RELY 1-study (Additional file 4). Non-responder analysis showed no difference for age (*p* = 0.65), but greater number of females among non-responders (*p* = 0.02). Twelve of 19 psychiatrists performed interviews, all performed IFAP ratings. Table 2 describes psychiatrists and patients.

**Table 2:** Characteristics of psychiatrists and patients**.** Characteristics of psychiatrists and patients, including the main diagnoses of the patients’ mental disorder(s) with impact on work capacity. In RELY 1 (RELY 2), six (seven) patients had been assigned two main diagnoses

	RELY 1	RELY 2
**Psychiatrists,** RELY 1: n=19^a^; RELY 2: n=35^b^		
Age		
31–40/ 41–50/ 51–60/ > 60 years/ missing	5/ 42/ 21/ 32/ 0%^c^	3/ 40/ 31/ 20/ 6%
Gender		
male	79%	83%
Experience		
Years since board certification as psychiatrist, mean (SD)	15.6 (9.7)	15.8 (9.0)
Number of years performing disability evaluations, mean (SD)	13.8 (9.2)	12.4 (7.5)
Number of evaluations in the previous year,		
0–4/ 5–20/ 21–50/ > 50/ missing	0/ 10 / 32 / 58/ 0%	6/ 17/ 31/ 40/ 6%
Time span from training to rating in days, mean (range)	404 days (115–578)	41 days (5–88)
**Patients,** RELY 1: n=30; RELY 2: n=40		
Age, years: mean (SD)	47.2 (8.6)	48.6 (10.1)
Gender		
male	57%	53%
Marital status		
Unmarried/ married/ divorced/ missing	20/ 40/ 40/ 0%	20/ 28/ 45/ 8%
Nationality		
Swiss/ others/ missing	63/ 23/ 14%	70/ 28/ 2%
Country of birth		
Switzerland/ others/ missing	67/ 27/ 6%	75/ 23/ 2%
Severity of disorder^d^		
mean (SD)	5.3 (2.1)	4.9 (1.8)
Typicality of study patient compared to other patients seen by the expert		
frequent / semifrequent / rare	36/ 44/ 20%	27/ 56/ 17%
**Main diagnoses** (ICD 10 classification) Number of diagnoses RELY 1: n=36; RELY 2: n=47		
Mood disorders (F3)	26%	40%
Neurotic, stress-related, somatoform disorders (F4)	19%	21%
thereof somatoform disorders (F45)	6%	15%
Organic (F0)	11%	9%
Disorders of adult personality and behaviour (F6)	11%	6%
Psychoactive substance use (F1)	3%	0%
Mental retardation (F7)	0%	2%
Behavioural and emotional disorders with onset in childhood (F9)	0%	2%
Patients without main diagnosis	19%	19%

a) Twelve out of 19 psychiatrists performed interviews, all performed ratings. b) Eleven out of 35 psychiatrists performed interviews, all performed ratings. c) Percentages are rounded to nearest whole numbers, d) Scale from 0 to 10, higher score indicates more severe disorder

**Fig. 1:**
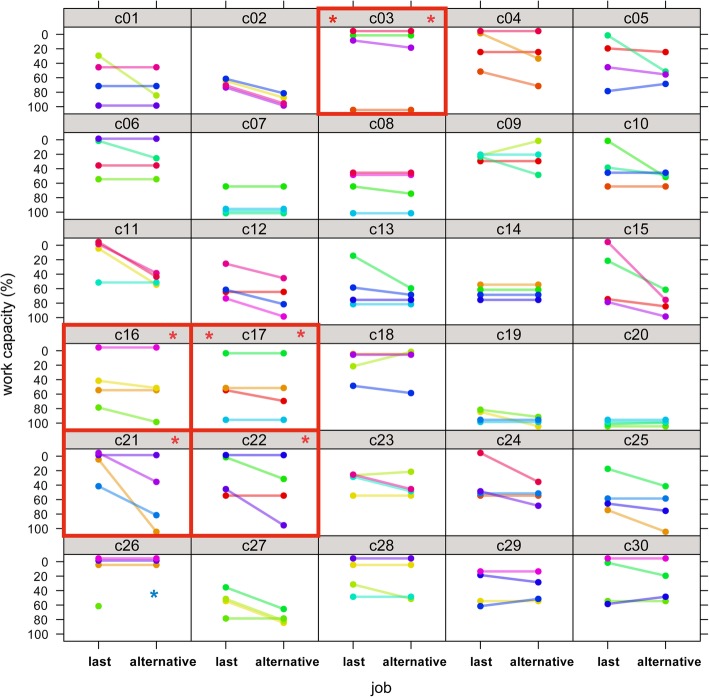
Work capacity ratings in RELY 1**.** Thirty plots of the four psychiatrists’ ratings of the patients’ overall work capacity in their last job and in alternative work for 30 patients (c01 to c30). The dots on the left in each cell indicate the psychiatrists’ ratings in relation to the patients’ last job and the dots on the right indicate their ratings in relation to the patients’ alternative work. The lines linking the dots represent the changes in the psychiatrists’ ratings. Each psychiatrist has a different colour. Red frames: psychiatrists disagreed with each other by 100% about the extent of work capacity. This was the case for two patients in relation to their last job, and for five patients in relation to alternative work.  Patients with maximum divergent expert ratings.  For ‘alternative work’, one rating of patient 26 was excluded from the analysis due to a violation of the rating rules

Mean WC was 43.6% for ‘last job’ (95%CI 34.1–53.2%) and 55.0% for ‘alternative work’ (95%CI 47.3–62.8%). When judging WC for ‘last job’ and ‘alternative work’, experts arrived at maximum divergent estimates in two (2/30, 6.7%) and five (5/30, 16.7%) patients, respectively. Although the WC ratings of the same patient varied widely across psychiatrists (Fig. 1), psychiatrists were highly certain that their own ratings reflected patients’ WC (rating scale: 7.4 points, mean, 95%CI 6.8–8.0 for ‘last job’ and 7.2 points, 95%CI 6.6–7.8 for ‘alternative work’). The ratings for ‘last job’ showed that some psychiatrists were systematically stricter than others (rater effect, ‘last job’ *p* < 0.001, ‘alternative work’ *p* = 0.07).

### Reliability and agreement

Table 3 provides variance estimates on the absolute and relative contributions of three sources of variation - psychiatrists, patients, residuals - to WC ratings, adding up to a total variance of 1092 (‘last job’) and 1060 (‘alternative work’), respectively. Inter-rater reliability on WC ratings was poor for ‘last job’ (ICC 0.38; 95%CI 0.19–0.55) and fair for ‘alternative work’ (ICC 0.43; 95%CI 0.22–0.60).

**Table 3:** Reliability and agreement measures**.** Absolute and relative contributions of the different sources of variation to work capacity ratings: work capacity ratings, total variance and variance components (psychiatrists, patients, residuals), reliability and agreement parameters for ‘last job’ and ‘alternative work’ in RELY 1 and RELY 2

Reference for WC		WC Mean (95%CI)	Total variance	Variance components Absolute variance (Relative variance)			Reliability	Agreement		
								Proportion of WC ratings between two psychiatrists whose ratings differed equal or less than the ‘maximum acceptable difference’ of 25 percentage points WC	‘Standard error of measurement’ (95%CI)	‘Maximum acceptable difference’ (95%CI)
				Psychiatrists	Patients	Residuals	ICC_abs.agree_ (95%CI)		reported in natural units	reported in natural units
Last job	RELY 1 *N* = 120	43.6% (34.1–53.2)	1092	263 (24%)	414 (38%)	415 (38%)	0.38 (0.19–0.55)	52.2% (94/180)	26.0% WC (21.5–31.0)	72.2% WC (59.5–86.0)
	RELY 2 *N* = 160	46.3% (39.9–52.6)	1064	76 (7%)	495 (47%)	493 (46%)	0.47 (0.29–0.61)	61.7% (148/240)	23.9% WC (20.8–27.0)	66.1% WC (57.7–74.9)
Alternative work	RELY 1 *N* = 119	55.0% (47.3–62.8)	1060	88 (8%)	457 (43%)	515 (49%)	0.43 (0.22–0.60)	61.6% (112/177)	24.6% WC (20.9–28.4)	68.1% WC (57.9–78.8)
	RELY 2 *N* = 155	62.9% (57.7–68.0)	669	50 (7%)	292 (44%)	328 (49%)	0.44 (0.25–0.59)	73.6% (170/231)	19.4% WC (16.9–22.0)	53.8% WC (46.8–61.0)

Legend: *WC*: work capacity, *% WC* = absolute percentage points in work capacity, *ICC*_*abs.agree*_ = intraclass correlation coefficient (agreement variant); CI: confidence interval

**Fig. 2:**
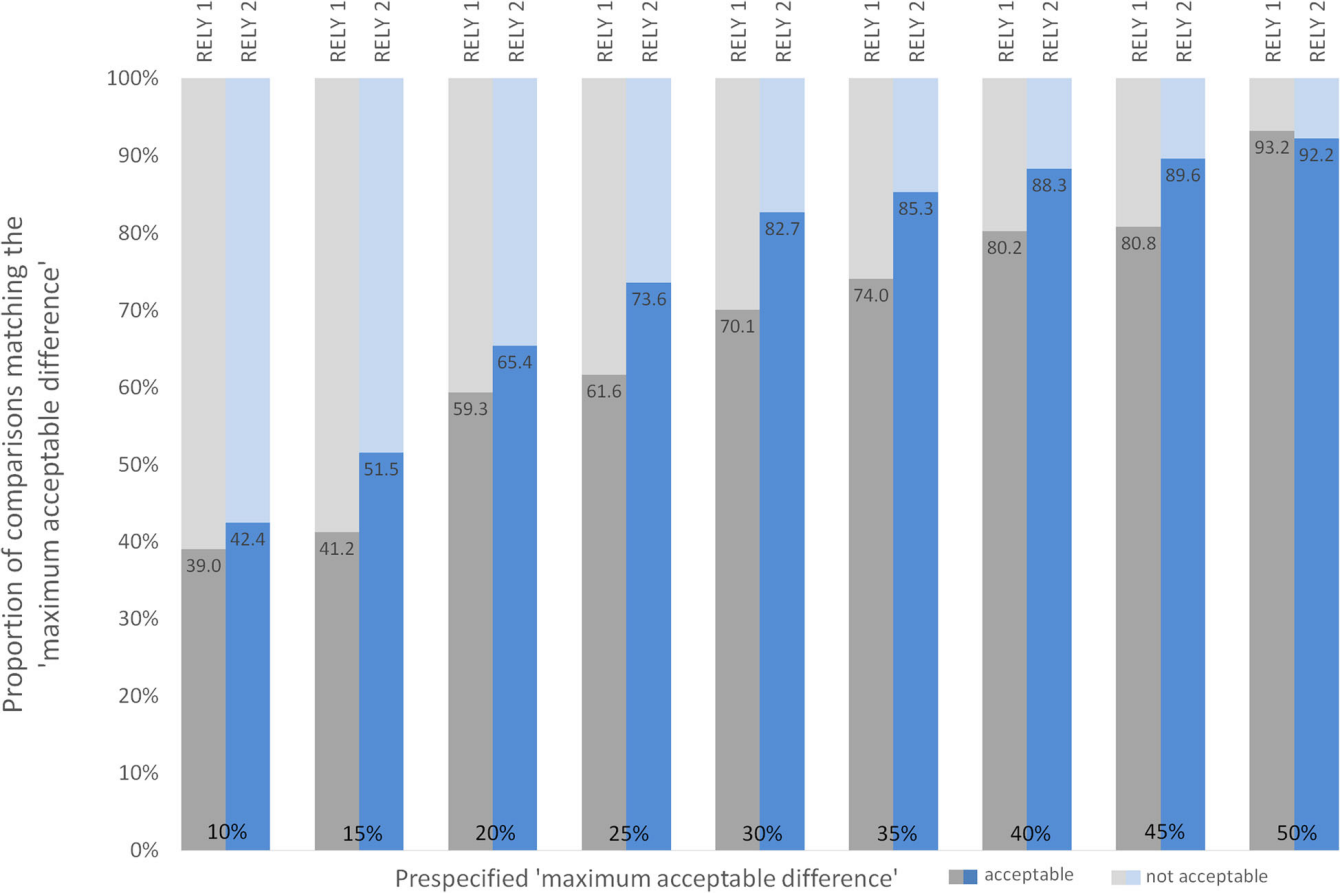
Agreement between experts for varying levels of ‘maximum acceptable difference’ This figure demonstrates the impact of varying limits for ‘maximum acceptable difference’ in WC ratings on level of agreement. Agreement is defined as the proportions of comparisons (in percentage, values in the bars) for whom the WC ratings between any two experts’ differ less than a prespecified limit, here, the ‘maximum acceptable agreement’. We used the expectations from a recent survey among stakeholders to specify the limits for ‘maximum acceptable difference’ (see Table 1 [6]). Illustrative examples from the stakeholder survey [6]. **a** Treating and expert psychiatrists defined 25 percentage points* in work capacity ratings between two experts as the ‘maximum acceptable difference’. In RELY 1, 61.6% (109/177) of comparisons would fall within this limit versus 73.6% (170/231) of comparisons in RELY 2. **b** Lawyers, judges and insurers defined 20 percentage points* in work capacity ratings between two experts as the ‘maximum acceptable difference’. In RELY 1, 59.3% (105/177) of comparisons would fall within this limit versus 65.4% (151/231) of comparisons in RELY 2. * upper limit of the interquartile range (see Table 1)

Figure 2 shows the proportion of psychiatrist-by-psychiatrist comparisons across a spectrum of varying limits for ‘maximum acceptable difference’ in WC between two psychiatrists. With a difference of < 25 percentage points WC – the limit suggested by treating psychiatrists and experts (Table 1) -, 61.6% of comparisons would fall within this prespecified limit.

Observed ‘standard error of measurement’ as a measure for agreement on WC was 26.0 percentage points (95%CI 21.5–31.0) for ‘last job’ and 24.6 percentage points (95%CI 20.9–28.4) for ‘alternative work’. Both results were larger than the expected ‘standard error of measurement’ converted from the ‘maximum acceptable difference’ that stakeholders considered appropriate (9.0 for experts and psychiatrists; 7.2 for lawyers, judges, insurers, Table 4).

**Table 4:** Expected versus observed agreement

a) Expected by stakeholders		b) Observed in the RELY studies			
‘Maximum acceptable difference’^a^	Corresponding ‘Standard error of measurement’			‘Standard error of measurement’	Corresponding ‘Maximum acceptable difference’
25% WC	9.0% WC	Last job	RELY 1	26.0% WC	72.2% WC
20% WC	7.2% WC		RELY 2	23.9% WC	66.1% WC
15% WC	5.4% WC	Alternative job	RELY 1	24.6% WC	68.1% WC
10% WC	3.6% WC		RELY 2	19.4% WC	53.9% WC

Legend: % WC = absolute percentage points in work capacity

^a^ derived from the stakeholder survey (Table 1) [6]

This table compares the expectations of Swiss stakeholders of the agreement in WC ratings between two experts, expressed as ‘maximum acceptable difference^a^’, with the agreement observed in the RELY studies, i.e., the variation between experts, expressed as ‘standard error of measurement’. Converting ‘maximum acceptable difference’ into ‘standard error of measurement’ and vice versa allows comparison of the level of agreement

a) Agreement expected by stakeholders: Treating and expert psychiatrists considered a difference of 25% WC between two experts as the ‘maximum acceptable difference’ (i.e. for example, expert A: 60% WC; expert B: 35% WC or 85% WC) which corresponds to a variation between experts of 9.0% WC ‘standard error of measurement’

If the ‘maximum acceptable difference’ between two experts were only 15% WC (i.e. for example, expert A: 60% WC, expert B: 45% WC or 75% WC), the corresponding variation between experts would be as low as 5.4% WC ‘standard error of measurement’

b) Agreement observed in the RELY studies: RELY 2_last job_ found a level of agreement of 23.9% WC ‘standard error of measurement’ which corresponds to a (‘maximum acceptable’) difference in WC of 66.1% (i.e. for example, expert A: 30% WC; expert B: 96% WC)

### RELY 2-study

Of 147 potentially eligible patients, 123 met inclusion criteria and 25 entered the RELY 2-study, along with 15 RELY 1-patient videos (Additional file 5). Non-responder analysis showed no difference for age (*p* = 0.09) or gender (*p* = 0.34). Twenty-four new psychiatrists participated in the study. Eleven RELY 1-psychiatrists performed the interviews, and all psychiatrists performed IFAP ratings. Table 2 provides characteristics of psychiatrists and patients.

**Fig. 3:**
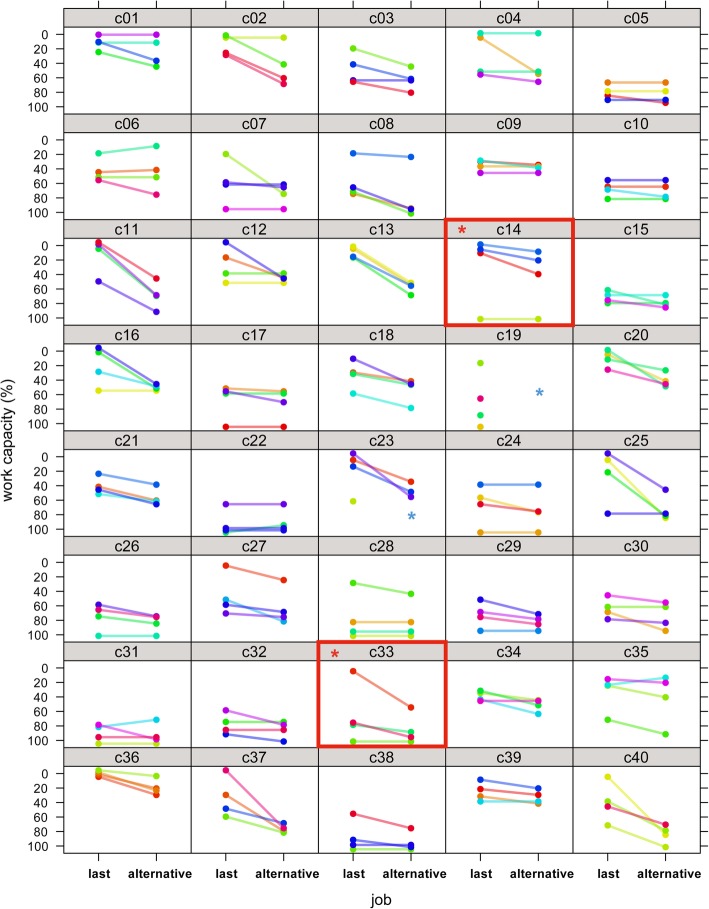
Work capacity ratings in RELY 2**.** Forty plots of the four psychiatrists’ ratings of the patients’ overall work capacity in their last job and in alternative work for 40 patients (c01 to c40). Red frames: Psychiatrists disagreed with each other by 100% about the extent of work capacity for two patients in their last job, and for no patient in relation to alternative work, which was the primary outcome.  Patients with maximum divergent ratings.  For ‘alternative work’, all ratings of patient 19 and one rating of patient 23 were excluded from the analysis due to violations of the rating rules

Mean WC was 46.3% for ‘last job’ (95%CI 39.9–52.6%) and 62.9% for ‘alternative work’ (95%CI 57.7–68.0%). Psychiatrists arrived at maximum divergent WC ratings in two patients for ‘last job’ (2/40, 5%) and none for ‘alternative work’. Again, WC ratings of the same patient varied widely across psychiatrists (Fig. 3), even though the psychiatrists were highly confident in their own ratings (rating scale: 7.7 points, mean, 95%CI 7.3–8.1 for ‘last job’ and 7.4 points, 95%CI 7.0–7.9, for ‘alternative work’). There was no rater effect (‘last job’, *p* = 0.07, ‘alternative work’, *p* = 0.10).

### Reliability and agreement

Table 3 provides variance estimates on the contributions of the different sources of variance to WC ratings, adding up to a total variance of 1064 (‘last job’) and 669 (‘alternative work’), respectively. Inter-rater reliability on WC (Table 3) was fair for ‘last job’ (ICC_abs.agree_ 0.47; 95%CI 0.29–0.61) and for ‘alternative work’ (0.44; 95%CI 0.25–0.59).

Figure 2 shows the proportion of psychiatrist-by-psychiatrist comparisons with difference in WC rating < 25 percentage points. Here, 73.6% of comparisons would fall within this limit. ‘Standard error of measurement’ was a difference in WC of 23.9 percentage points (95%CI 20.8–27.0) for ‘last job’ and of 19.4 percentage points (95%CI 16.9–22.0) for ‘alternative work’. Both results were larger than the expected ‘standard error of measurement’ converted from the ‘maximum acceptable difference’ that stakeholders considered appropriate (9.0 for experts and psychiatrists, 7.2 for lawyers, judges, insurers, Table 4).

### Comparing RELY 1 and 2

#### Sociodemographics

Psychiatrists and patients in RELY 1 resembled those in RELY 2. RELY 1 and 2 patients showed no difference in WC_last.job_ (43.6% versus 46.3%, 2.7% WC, mean difference, 95%CI − 8.8 to 13.9), but a trend for higher WC_alternative.work_ (55.0% versus 62.9%, 7.9% WC, 95%CI − 1.1 to 17.1) in RELY 2.

#### Variances, reliability, and agreement (Table 3)

While 24% of variance in WC for ‘last job’ in RELY 1 was attributable to the psychiatrists, more intensive standardisation reduced this variation to 7% in RELY 2. The reliability of the expert judgement on the patients’ WC did not change in any of the four situations (RELY 1 and 2, last job and alternative work), indicating that our training programme in functional evaluation did not improve the reliability of expert judgements (ICC_abs.agree_ between 0.38 [poor] and 0.47 [fair]), i.e., experts were not enabled to better distinguish between individuals with higher and those with low remaining WC.

With regards to agreement between experts, the proportion of psychiatrist-by-psychiatrist comparisons that stayed below the prespecified threshold was higher in RELY 2 for all thresholds (Fig. 2). For example, at a threshold of 25 percentage points WC, the proportion of comparisons within the ‘maximum acceptable difference’ was 73.6% in RELY 2, contrasted by 61.6% in RELY 1 (*p* = 0.008). The comparison of SEM_alternative.work_ showed a significant change by − 5.2 percentage points (95%CI − 9.7 to − 0.6, Tables 3 and 4) in RELY 2.

#### Patients’ and psychiatrists’ perception of the functional evaluation

Patients’ approval of the functional evaluation was high, with scores of 8.0 points (mean, 95%CI 7.2–8.8) in RELY 1 and 9.4 (95%CI 9.1–9.7) in RELY 2 for ‘Overall perception of fairness’ (see Additional file 3). Psychiatrists experienced the functional evaluation as a valuable addition to their current approach. RELY 2-psychiatrists reported a greater focus on functional aspects (21/25, 84%) by integrating the IFAP in their WC evaluations and acknowledged substantial professional benefit from the training (96%, 24/25).

## Discussion

### Main findings

Two multi-centre real-life reproducibility studies (RELY 1 and 2) of expert psychiatrists assessing WC in patients with mental disorders found that more intensive training in functional evaluation of WC reduced variance but did not change psychiatrists’ low ability to discriminate patients with different degrees of WC for alternative work from each other. Post-hoc comparisons of RELY 1 and 2 indicated that intensive training achieved higher agreement between experts for WC_alternative.work_ ratings, albeit improvements fell short of expectations. Patients perceived the functional evaluation as fair, and psychiatrists perceived it as an useful addition to their current practice of work disability evaluation.

### Strengths, limitations, challenges in design and performance

Strengths of our studies include the use of real-life disability evaluations with their heterogeneous mix of typical patients, a broad spectrum of experts, and calibration of experts and description of work demands as reference. Despite clear differences in concepts [12, 13, 25], both reproducibility parameters ‘reliability’ and ‘agreement’ are frequently used interchangeably in the literature. In our study, we analysed these parameters separately.

We did not achieve the expected improvement in reliability in RELY 2_alternative.work_. There, experts considered fewer patients as fully able or fully unable to work in alternative work compared to RELY 1, and consequently, almost all patients were attributed some remaining WC. The reduction of patient variance in RELY 2_alternative.work_ indicates that patients were perceived as more homogeneous than those in RELY 1. However, the equal reduction of variance across all variance components resulted in unaltered low discrimination of remaining WC across patients (ICC_alternative.work_ RELY 1 versus RELY 2: 0.43 versus 0.44, Table 5) [29]. This reflects reality: ‘It *is* more difficult to tell people apart if they are relatively similar than if they are very different’([25], Chapter 8).

**Table 5:** Interaction of various sources of variance on reliability

Illustration of the interaction of various sources of variance and their impact on the reliability measure ICC.	
**General formula for ICC**_***abs.agree***_ [25]: $\frac{\sigma_{Patients}^2}{\sigma_{Patients}^2+{\sigma}_{Psychiatrists}^2+{\sigma}_{Residuals}^2\ }$	
**Example 1 - Analogy to the situation observed in RELY 1**: the ICC is calculated based on a patient variance of 500, a psychiatrist variance of 100 and a large residual (unexplained) variance of 500. ICC = $\frac{\mathbf{500}}{\mathbf{500}+100+500} = 0.45$ which corresponds to a fair discrimination of patients [26]	
**Example 2 - Analogy to the situation observed in RELY 2**: The ICC is calculated with a patient variance of 250, a psychiatrist variance of 50 and a large residual (unexplained) variance of 250. ICC = $\frac{\mathbf{250}}{\mathbf{250}+50+250} = 0.45$ which corresponds to a fair discrimination of patients (equal to example 1)	
Despite reduction of total variance, the proportionate reduction of variance across all sources of variance results in an ICC of 0.45 identical to example 1. Despite reduction of variance by half, the ability to discriminate patients in their ability to work did not change.	
**Example 3 - Typical situation for a reliable instrument**: Most variance is explained by patient variance, with little psychiatrist variance and residual variance: patient variance of 500, psychiatrist variance of 25, and residual variance of 75. As a result, expert variance and residual variance contribute little to the total variance, indicating low measurement error. This allows excellent discrimination among patients. ICC = $\frac{\mathbf{500}}{\mathbf{500}+25+75} = 0.83$	

Agreement provides information about the measurement error of an instrument. Here, the ‘Instrument Functional Evaluation’ were experts with their presumed ability to elicit the relevant information from patients, experts who have suitable instruments, a good understanding of work demands, and skills to turn the compiled information into reasoned judgement on WC. Intensive manualised training improved expert agreement, but agreement remained low, indicating that the measurement error of functional evaluation with limited and intensive training passed any maximum acceptable disagreement [12]. The high measurement error far exceeding patient variance contributed directly to low reliability (Streiner 2014, chapter 8 [25]).

Studies on WC evaluations focus on reproducibility without addressing validity [8]. Although validity is crucial for credibility, it remains challenging to quantify work (in-)capacity, a social notion with implicit societal values, using psychometric methodology. Professional consensus grounded in evidence or predictive validity may provide a surrogate for validity. This assumption needs proof.

Psychiatrists constantly rated their confidence in their own WC assessment as very high, despite the fact that experts seeing the same patient often disagreed with each other. This phenomenon suggests that individual raters are working with different frames of reference, as can be seen with chronic pain: Some clinicians believe strongly that patients with (for example) fibromyalgia will not be able to work, while others feel very differently.

Prior evidence to inform our study design was very limited [7, 8]: We lacked information about potential effect sizes, sources and extent of variations, the impact of expert calibration on reproducibility, criteria to decide on the most appropriate outcome measure, trustworthy data to feed the design, including power calculation for reliability and agreement estimates.

The original research plan proposed a reliability study on functional evaluation and WC judgements (RELY 1), followed by a randomised comparison with current practice [16]. Since governmental changes stalled RELY 1 for more than a year, the observed reproducibility reflects the impact of short training in functional evaluation without standardisation [7]. The reproducibility of experts without training may be comparably low or worse.

External factors interfered with the planned randomised comparison. It remains untested whether patients and lawyers would have indeed consented to a chance -rather than a ‘preferred option’- allocation to either type of disability evaluation. In case of objections, a non-randomised comparison might have been the best alternative to test the effectiveness of training on reproducibility. Since the RELY studies lacked randomisation (‘low quality evidence’), findings need to be interpreted with caution.

Some might argue that our study examines videos of patients rather than actual patients. However, our design purposefully mimics real-life disability assessments (see Bachmann 2016 [11], Fig. 3; [8]) where training intensity was balanced against feasibility for practicing psychiatrists, and the functional evaluation was integrated in individual conventional psychiatric interviews. Semi-structured questions introduced mandatory themes about work, but left space for open questions. However, these elements facilitate heterogeneity in the raters’ interpretation and reduced the intended reproducibility. This contrasts lab-like designs with highly standardised video-recorded interviews and experienced interviewers calibrated over longer periods in performing and rating interviews which achieve high reproducibility [8], but do not mirror reality.

Our study focused on the psychiatrists’ evaluations all of which were part of multi-disciplinary WC evaluations. The ultimate judgments of remaining work capacity would have to integrate functional and WC evaluations from other (e.g., musculoskeletal, neurological) disciplines which adds challenges that were beyond our study.

The complexity of WC evaluation brings about many more sources of variation than we could address in our study (Table 6) [5, 30]. We targeted the study to raise a low ICC (around 0.4) to a fair to good level (ICC of 0.6). Systematic efforts will be required to identify and tackle additional modifiable sources of variance in future research.

**Table 6:** Sources of variation. Potential factors for the three sources of variation (psychiatrists, patients, residuals) which may contribute to the variance in overall WC ratings (modified from [5, 30])

Source of variation	Factors that may impact on the variance of overall work capacity
Psychiatrists	• Experience in disability evaluation • Knowledge about previous work • Structuring and prioritizing of information • Psychiatrists’ idiosyncrasies (e.g. leniency/strictness)
Patients	• Socio-demographic features • Diagnosis, severity of disorder • Compliance, including malingering • Skills in presenting their case • Symptom exaggeration
Residuals	• Interaction psychiatrists*patients • Interaction patient*last job; patient*‘alternative work’
	External factors: • Changes in legislation with impact on medical evaluations • Interferences of legal demands with medical judgements • Turn-over of staff involved in the studies • Overall attitude in society towards disability

### Comparisons to other studies

Systematic research on direct evaluation of WC is sparse [7]. Our recent systematic review with a low threshold for inclusion identified 16 reproducibility studies from 12 countries published over a period of 25 years [8]. Most studies were of low methodological quality, only three studies were conducted with real patients, and most reported only poor to fair reproducibility for work disability. Though, exceptions existed [31, 32].

### Implications for practice and policy

Was training in functional evaluation sufficient to tackle the tasks as medical expert? A critical revision would include a review of training material, training intensity, and duration, and documention of success in expert calibration. This requirement is analogous to medical training where trainees acquire skills, e.g. in ultrasound imaging, by performing hundreds of scans under supervision in order to distinguish normal images from pathologies and discriminate similar but different pathologies.

Current expert-based WC evaluations contain many discretionary judgements that contribute to low reproducibility. Standardising the process as done in RELY 2 appears to have some but not sufficient impact. Experts have called for more tools to complement their functional judgements [33], such as the Work Disability-Functional Assessment Battery (WD-FAB [34, 35]) that elicits self-reported behavioural and physical impairments, or tests for mental or physical functional capacities [36]. The impact of these tests on the experts’ final judgement and their agreement on WC would require empirical testing.

More far-reaching approaches would restrict the physicians’ role to their professional core competences: reporting the impact of impaired health on the patients’ functional capacities. Work capacity is legally defined as expected earning capacity in suitable work [37]. Most physicians understand little about the diversity of modern work life, specific job demands, and their interactions with functional impairments. These tasks could be shifted to labour experts and their specific expertise. Models exist in the Netherlands [38], where medical experts establish the patients’ functional profile and labour experts match potential jobs for determining wage replacement. In Sweden and Denmark, labour experts participate in interdisciplinary evaluation teams [39].

What level of variation is acceptable for WC evaluation (Figs. 1, 3, Table 1)? Insurers who commission evaluations expressed lowest tolerance for ‘maximum acceptable differences’ between experts, while psychiatrists who perform the evaluations showed the highest tolerance [6], albeit tolerance was half the variation observed in RELY. While crucial to get variation down, it is equally important for insurers to align their expectations with reality and abandon prospects on the precision in WC ratings that evaluations are unlikely to provide even with improved methods.

Acceptable level of variation in WC evaluation is a social policy issue that requires a societal discussion, adressing the balance between the principles of fairness (‘similar treatment for similar cases’) versus the principle of treating each case individually – implying discretionary expert judgements and highly variable WC ratings across cases. Our stakeholder survey demonstrates a strong preference for fair and equal evaluations.

What level of agreement would be required to reach these objectives? Widely accepted guidance for evaluating psychological tests [40] require reliabilities of 0.9 for decisions on individuals. In contrast, clinical guidance acknowledges that purpose and consequences of scores determine how much error should be allowed in clinical decision-making [25]. While the functional evaluation uses instruments such as IFAP to ascertain the patients’ functional capacities, the translation from functional capacities to WC is a judgement at the experts’ discretion, not a measurement. Judgements will never reach the same level of reliability and agreement as fully standardised psychological tests do. Nevertheless, society needs a discussion on the desired levels, how to get there and at what price.

### Future research

Unexplained variance remained a major concern in the RELY studies. Research needs to identify additional potentially modifiable sources of variance (Streiner 2014, chapter 8 [25]), such as the psychiatrist*patient interaction [5, 8, 12, 30] and may require lab-type settings [31, 32]. Reproducibility is closely linked to the population under investigation and its characteristics [13], (Streiner 2014, chapter 8 [25]). To reach an in-depth understanding of the performance of functional evaluation in social security, similar studies need to investigate other health conditions and settings. Despite current low reproducibility which badly affects validity, considerations on how to establish validity of WC evaluations beyond professional consensus are warrented.

All aspects of WC evaluations are seriously under-researched which challenges the planning of studies. We need data about potential effect sizes, sources and extent of variations, impact of expert calibration on reproducibility, criteria to decide on outcome measure, data to feed power calculations. Training experts alone may not result in acceptable reproducibility. Nevertheless, a better understanding of the cognitive approaches how medical experts come up with WC ratings may inform training. Furthermore, teaching ‘functional evaluation’, a novel technique, needs iterative refinement integrating experience from practice into training curricula, including material, intensity, duration, teaching techniques, and evaluation of learning.

No study in our systematic review [8] provided recommendations on what level of reproducibility would be mandatory, desirable, or acceptable to ensure equal treatment of patients. A societal discussion would need to address alternative approaches with their advantages and limitations, how to get there and at what cost. Conceptually, to establish a direct link from functional capacity to WC would require to match ICF-based [41] functional profiles of patients with ICF-based functional features of job demands in today’s working environment. The dimension of the task may require the umbrella of organisations such as World Health Organisation or International Social Security Association [1].

## Conclusions

Evidence from non-randomised studies suggests that intensive training in functional evaluation may increase agreement on WC between experts, but fell short to reach stakeholders’ expectations. It did not alter reliability. Isolated efforts in training psychiatrists may not suffice to tackle the complexity of the task to reach the expected level of agreement. Adding additional components in the procedures of WC evaluations may deserve considerations.

## Additional files

Additional file 1: Planned design versus actual conduct of the studies**.** Comparison of the design for the RELY study as planned with the actual conduct of the studies as RELY 1 and RELY 2. *SIM = Swiss Insurance Medicine, professional society of medical experts (DOCX 15 kb)

Additional file 2: Design of the RELY studies**.** Both RELY studies recruited psychiatrists from the same population (practicing experts being SIM members). Training differed in training intensity and duration to implementation. Patients were recruited from the same population through the National Disability Insurer and Suva. In RELY 2, we re-used 15 interviews from RELY 1. The 25 new RELY 2-interviews were conducted by 11 RELY 1-interviewers who were re-trained for rating. Both studies used the same implementation procedure. (PNG 45 kb)

Additional file 3: Questionnaire on Perceived Fairness. Patients’ perception of the fairness of the work disability evaluation. The questionnaire had 29 items on a scale from 1 to 5 (higher scores indicate stronger affirmation) and a single item on overall perception of fairness on a scale from 10 to 0. The table shows five typical items. (DOCX 18 kb)

Additional file 4: Patient flow in RELY 1**.** * *n* = 1 missing due to violation of rating rules (JPG 67 kb)

Additional file 5: Patient flow in RELY 2**.** *: *n* = 5 missing due to violation of rating rules (JPG 74 kb)

## Data Availability

The datasets used and analysed during the current study are available from the corresponding author on reasonable request.

## Abbreviations

95%CI: 95% confidence interval
GRRAS: Guidelines for Reporting Reliability and Agreement Studies
ICC: Intraclass correlation coefficient
ICD 10: International Classification of Diseases (10th revision)
ICF: International Classification of Functioning, Disability and Health
IFAP: Instrument for Functional Assessment in Psychiatry
OECD: Organisation for Economic Co-operation and Development
RELY: Reliable disability EvaLuation in psychiatrY
SMBA: Sociaal-Medische Beoordeling van Arbeidsvermogen (‘Socio-Medical Assessment of Work Capacity’)
Suva: Swiss National Accident Insurance Fund
WC: Work capacity

## Footnotes

1 The administrative changes of the Swiss Government led to the instalment of an electronic system for random distribution of patients to registered assessment centres www.suissemedap.ch and to the implementation of strict deadlines for the delivery of reports.

## References

[1] International Social Security Association I: Country Profiles. https://www.issa.int/en/country-profiles, last accessed 14.04.2019.

[2] OECD (2010). Sickness, disability and work: breaking the barriers. A synthesis of findings across OECD countries.

[3] Schandelmaier S, Fischer K, Mager R, Hoffmann-Richter U, Leibold A, Bachmann MS, Kedzia S, Jeger J, Marelli R, Kunz R (2013). Evaluation of work capacity in Switzerland: a survey among psychiatrists about practice and problems. Swiss Med Wkly.

[4] de Boer W, Brage S, Kunz R (2018). Insurance medicine in clinical epidemiological terms: A concept paper for discussion. Dutch J Occup Insurance Med (Tijdschrift voor Bedrijfs- en Verzekeringsgeneeskunde - TBV).

[5] Spanjer J, Krol B, Brouwer S, Groothoff JW (2010). Sources of variation in work disability assessment. Work.

[6] Schandelmaier S, Leibold A, Fischer K, Mager R, Hoffmann-Richter U, Bachmann MS, Kedzia S, Busse JW, Guyatt GH, Jeger J (2015). Attitudes towards evaluation of psychiatric disability claims: a survey of Swiss stakeholders. Swiss Med Wkly.

[7] Baumberg Geiger B, Garthwaite K, Warren J, Bambra C (2018). Assessing work disability for social security benefits: international models for the direct assessment of work capacity. Disabil Rehabil.

[8] Barth J, WELd B, Busse JW, Hoving JL, Kedzia S, Couban R, Fischer K, DYv A, Spanjer J, Kunz R (2017). Inter-rater agreement in evaluation of disability: systematic review of reproducibility studies. BMJ.

[9] Anner J, Kunz R, Wd B (2013). Reporting about disability evaluation in European countries. Disabil Rehabil.

[10] de Boer W, Marelli R, Hoffmann-Richter U, Eichhorn M, Jeger J, Colomb E, Mager R, Fischer K, Kunz R (2015). Functional assessment in psychiatry. The manual (die Funktionsorientierte Begutachtung in der Psychiatrie. Ein manual).

[11] Bachmann M, de Boer W, Schandelmaier S, Leibold A, Marelli R, Jeger J, Hoffmann-Richter U, Mager R, Schaad H, Zumbrunn T (2016). Use of a structured functional evaluation process for independent medical evaluations of claimants presenting with disabling mental illness: rationale and design for a multi-center reliability study. BMC Psychiatry.

[12] de Vet HC, Terwee CB, Knol DL, Bouter LM (2006). When to use agreement versus reliability measures. J Clin Epidemiol.

[13] Kottner J, Audigé L, Brorson S, Donner A, Gajewski BJ, Hróbjartsson A, Roberts C, Shoukri M, Streiner DL (2011). Guidelines for reporting reliability and agreement studies (GRRAS) were proposed. J Clin Epidemiol.

[14] World Report on Disability In*.* Geneva: World Health Organization; 2011. www.who.int/disabilities/world_report/2011/en/ last accessed 14.Apr.2019.

[15] Holwerda A, Groothoff JW, de Boer MR, van der Klink JJL, Brouwer S (2013). Work-ability assessment in young adults with disabilities applying for disability benefits. Disabil Rehabil.

[16] Kunz R: Improving reliability and transparency of Independent Medical Expertises (IMEs) and their usefulness to social judges, claimants and social insurance organisations. In*.*: Swiss National Science Foundation, SNSF; 2013. http://p3.snf.ch/project-144200 last accessed 14.Apr.2019.

[17] von Allmen DY, Kedzia S, Dettwiler R, Vogel N, Kunz R, de Boer W: Higher agreement in psychiatric disability evaluations through information about claimants' self-perceived work capacities and limitations (in preparation).

[18] Linden M, Baron S, Muschalla B (2009). Mini-ICF-APP. Mini-ICF-rating for activity and participation in mental health disorders.

[19] Crits-Christoph P, Johnson J, Gallop R, Gibbons MBC, Ring-Kurtz S, Hamilton JL, Tu X (2011). A generalizability theory analysis of group process ratings in the treatment of cocaine dependence. Psychother Res.

[20] Harmsen J (2013). Development and analysis of the questionnaire for client monitoring in social-medical affairs.

[21] Lohss R, Bachmann M, Wd B, Kunz R, Fischer K (2018). What are the concerns of claimants who underwent a disability assessment?. Dutch J Occup Insurance Med (Tijdschrift voor Bedrijfs- en Verzekeringsgeneeskunde - TBV).

[22] ICD-10. International Statistical Classification of Diseases and Related Health Problems 10th Revision [https://icd.who.int/browse10/2016/en, last accessed 19.Apr.2019].

[23] Bonett DG (2002). Sample size requirements for estimating intraclass correlations with desired precision. Stat Med.

[24] Schellart AJ, Mulders H, Steenbeek R, Anema JR, Kroneman H, Besseling J (2011). Inter-doctor variations in the assessment of functional incapacities by insurance physicians. BMC Public Health.

[25] Streiner DL, Norman GR, Cairney J. Health measurement scales: a practical guide to their development and use. Oxford: Oxford University Press; 2014.

[26] Fleiss JL (1981). Statisticals methods for rates and proportions.

[27] Satterthwaite FE (1946). An approximate distribution of estimates of variance components. Biom Bull.

[28] Sterne JA, Hernan MA, Reeves BC, Savovic J, Berkman ND, Viswanathan M, Henry D, Altman DG, Ansari MT, Boutron I (2016). ROBINS-I: a tool for assessing risk of bias in non-randomised studies of interventions. BMJ.

[29] Ten Cate DF, Luime JJ, Hazes JM, Jacobs JW, Landewe R (2010). Does the intraclass correlation coefficient always reliably express reliability? Comment on the article by Cheung et al. Arthritis Care Res (Hoboken).

[30] Kobak KA, Brown B, Sharp I, Levy-Mack H, Wells K, Okum F, Williams JBW (2009). Sources of unreliability in depression ratings. J Clin Psychopharmacol.

[31] Schellart AJM, Zwerver F, Anema JR, derBeek AJ V (2013). The influence of applying insurance medicine guidelines for depression on disability assessments. BMC Research Notes.

[32] Slebus FG, Kuijer PFM, Willems JHBM, Frings-Dresen MHW, Sluiter JK (2010). Work ability assessment in prolonged depressive illness. Occup Med (Lond).

[33] Kunz R, Verbel A, Weida R, Hoving JL, Weinbrenner S, Friberg E, De Boer WEL, Schaafsma F (2019). Knowledge and training needs on evidence-based medicine in social security and insurance medicine. An international survey.

[34] Marfeo EE, McDonough C, Ni P, Peterik K, Porcino J, Meterko M, Rasch E, Kazis L, Chan L (2019). Measuring work related physical and mental health function: updating the work disability functional assessment battery (WD-FAB) using item response theory. J Occup Environ Med.

[35] Meterko M, Marino M, Ni P, Marfeo E, McDonough CM, Jette A, Peterik K, Rasch E, Brandt DE, Chan L. Psychometric evaluation of the improved work-disability functional assessment battery. Arch Phys Med Rehabil epub. 2018. 10.1016/j.apmr.2018.09.125. 10.1016/j.apmr.2018.09.125 PMC8787773 30578775

[36] Gouttebarge V, Wind H, Kuijer PP, Frings-Dresen MH (2004). Reliability and validity of functional capacity evaluation methods: a systematic review with reference to Blankenship system, Ergos work simulator, ergo-kit and Isernhagen work system. Int Arch Occup Environ Health.

[37] de Boer WEL, Besseling JJM, Willems JHBM (2007). Organisation of disability evaluation in 15 countries. Revue pratiques et organisations des soins.

[38] Mabbett D (2003). Definitions of disability in Europe: a comparative analysis. In*.* Brussels: European Commission. Directorate for Employment and Social Affairs.

[39] Toren K, Jarvholm B (2015). Who is the expert for the evaluation of work ability?. Scand J Work Environ Health.

[40] (AERA) AERA, (APA) APA, (NCME) NCoMiE (2013). Standards for educational and psychological testing.

[41] World Health Organisation. International Classification of Functioning, Disability and Health. [http://www.who.int/classifications/icf/en/]. Last accessed: 14.04.2019

