# Supplementary material for: The reproducibility of psychiatric evaluations of work disability: two reliability and agreement studies
Source: BMC Psychiatry. 2019 Jul 3;19:205. doi: 10.1186/s12888-019-2171-y (PMC6607597; doi:10.1186/s12888-019-2171-y)
Supplement: Supplementary file 1 — Planned design versus actual conduct of the studies. Comparison of the design for the RELY study as planned with the actual conduct of the studies as RELY 1 and RELY 2. *SIM = Swiss Insurance Medicine, professional society of medical experts (DOCX 15 kb) [file 12888_2019_2171_MOESM1_ESM.docx]

|  | | **Protocol** |  | **RELY 1** | **RELY 2** |
| --- | --- | --- | --- | --- | --- |
| **Study design** | | Reliability study followed by a randomised trial that compares the functional evaluation with usual practice |  | Two reproducibility studies | |
|  |  |  |  | Comparison of RELY 1 (limited training) with RELY 2 (intensive training) | |
| **Recruitment** | **Patients** |  | Through the office of the National Disability Insurer and Suva | | |
|  | **Interviewing psychiatrists** |  | Through the assessment centers | | Psychiatrists from RELY 1 |
|  | **Rating psychiatrists** |  | Through the assessment centers  (members of SIM) | | Through the membership list from SIM* |
| **Training** | **Functional evaluation:**  **•** Interview  **•** IFAP rating | 9 hours of training were considered to be sufficient in producing fair to good reliability |  | 3x3 hours  of training | 4x3 and 1x6 hours of intensive training including comprehensive calibration of experts |
|  | **Training-to-rating delay** (mean) | Timely implementation |  | Delayed implementation  (404 days) | Timely implementation  (40 days) |
| **Data acquisition** | |  | IFAP through secured website | | |
| **Outcome measures** | |  | Primary outcome: WC in alternative work  Secondary outcomes: WC in the last job, experts’ certainty in judgements, patients’ and experts’ perception | | |
| **Data analysis** | |  | Reliability: ICC_absolute.agreement_  Agreement: a) Proportion of agreement using prespecified ‘maximum acceptable differences’ and b) ‘standard error of measurement’ | | |
