# Supplementary figures and images for: The reproducibility of psychiatric evaluations of work disability: two reliability and agreement studies
Source: BMC Psychiatry. 2019 Jul 3;19:205. doi: 10.1186/s12888-019-2171-y (PMC6607597; doi:10.1186/s12888-019-2171-y)

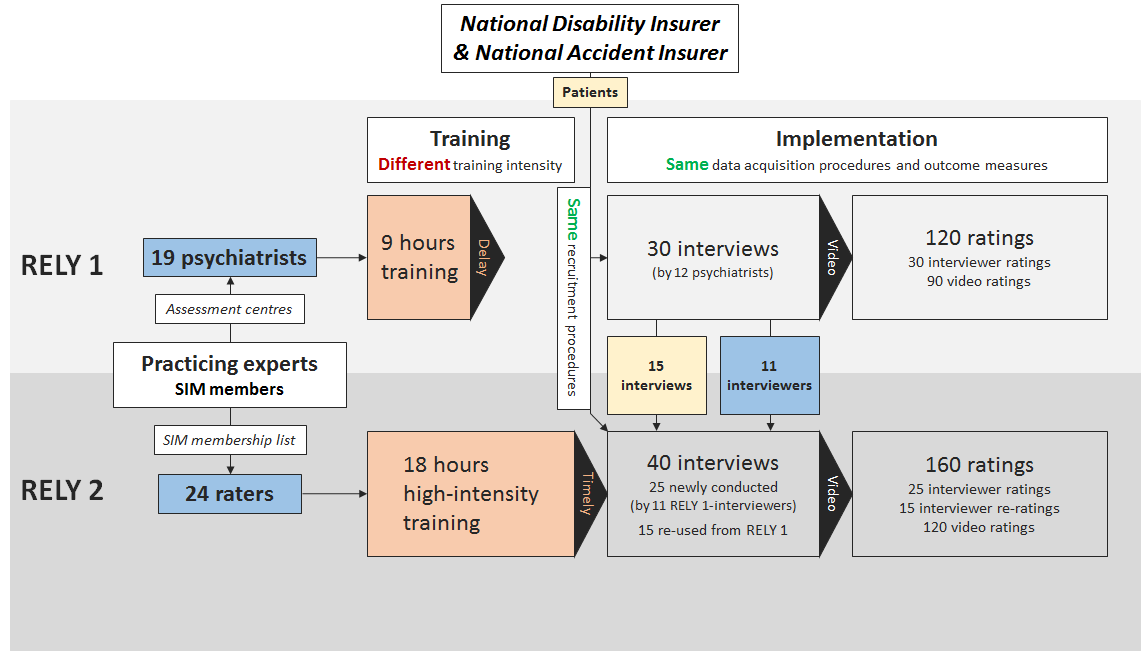

Supplement: Supplementary file 2 — Design of the RELY studies. Both RELY studies recruited psychiatrists from the same population (practicing experts being SIM members). Training differed in training intensity and duration to implementation. Patients were recruited from the same population through the National Disability Insurer and Suva. In RELY 2, we re-used 15 interviews from RELY 1. The 25 new RELY 2-interviews were conducted by 11 RELY 1-interviewers who were re-trained for rating. Both studies used the same implementation procedure. (PNG 45 kb) [file 12888_2019_2171_MOESM2_ESM.png]

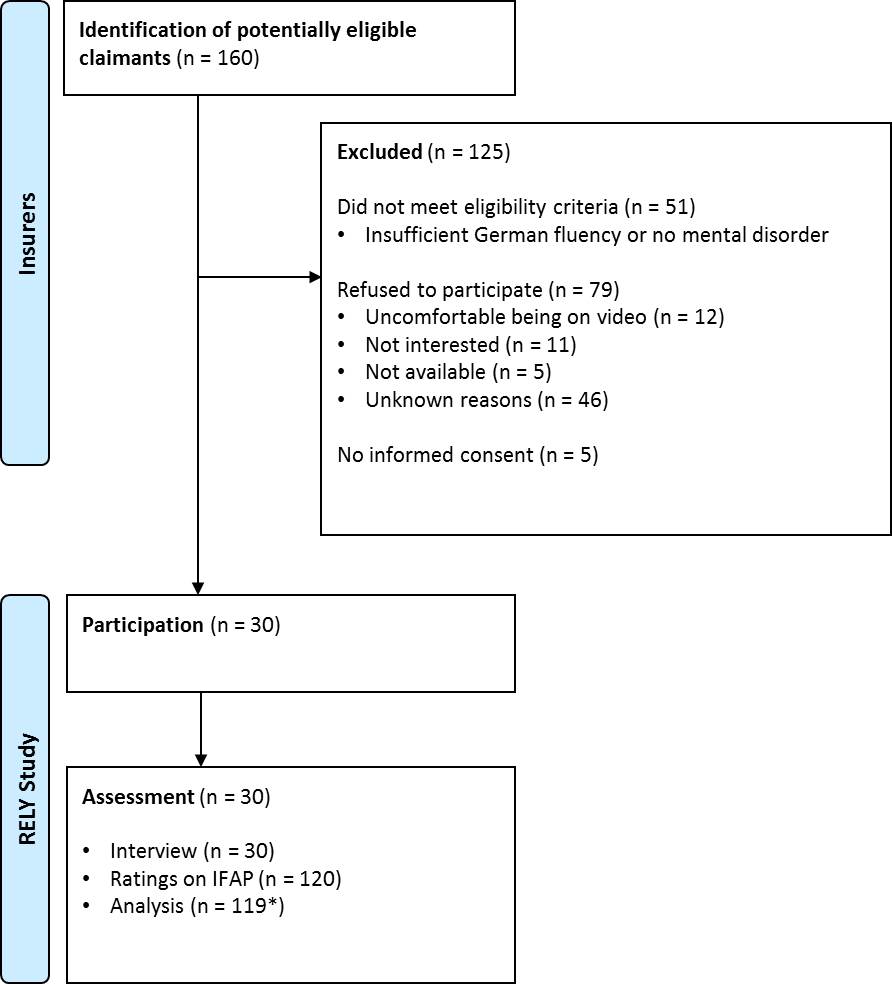

Supplement: Supplementary file 4 — Patient flow in RELY 1. * n = 1 missing due to violation of rating rules (JPG 67 kb) [file 12888_2019_2171_MOESM4_ESM.jpg]

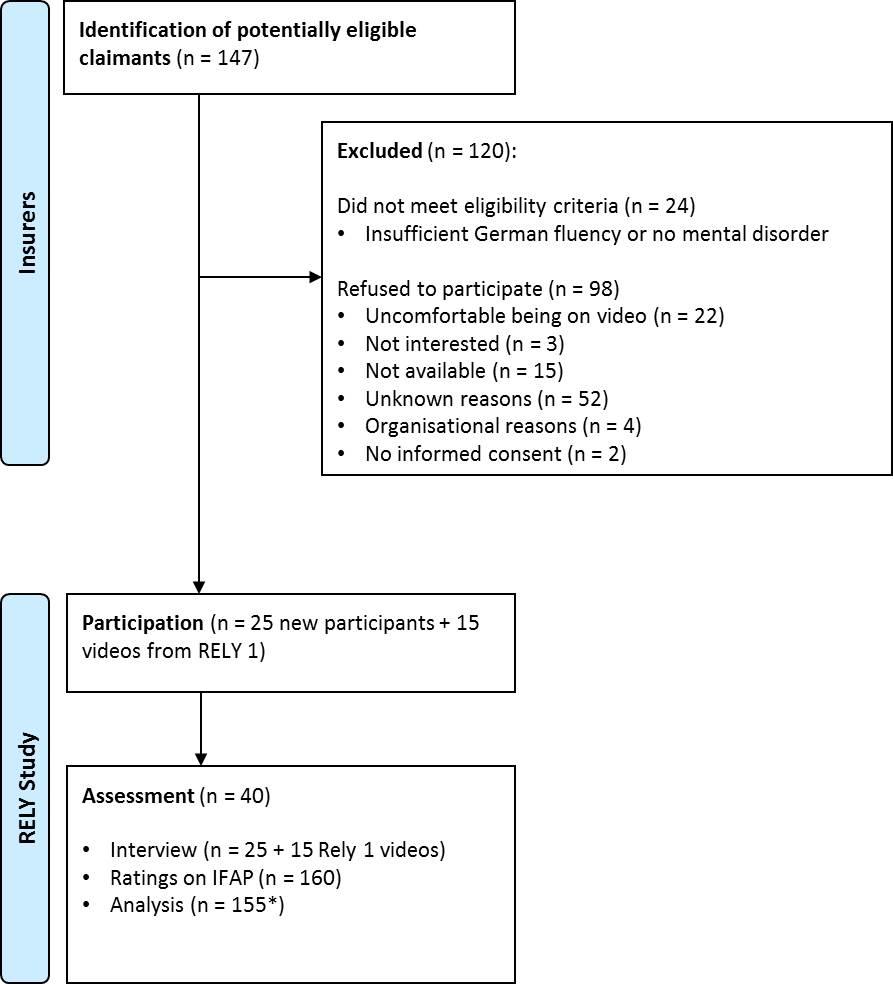

Supplement: Supplementary file 5 — Patient flow in RELY 2. *: n = 5 missing due to violation of rating rules (JPG 74 kb) [file 12888_2019_2171_MOESM5_ESM.jpg]
