# Supplementary material for: The reproducibility of psychiatric evaluations of work disability: two reliability and agreement studies
Source: BMC Psychiatry. 2019 Jul 3;19:205. doi: 10.1186/s12888-019-2171-y (PMC6607597; doi:10.1186/s12888-019-2171-y)
Supplement: Supplementary file 3 — Questionnaire on Perceived Fairness. Patients’ perception of the fairness of the work disability evaluation. The questionnaire had 29 items on a scale from 1 to 5 (higher scores indicate stronger affirmation) and a single item on overall perception of fairness on a scale from 10 to 0. The table shows five typical items. (DOCX 18 kb) [file 12888_2019_2171_MOESM3_ESM.docx]

| **Items of the Perceived Fairness Questionnaire** | **RELY 1** | | | **RELY 2** | |
| --- | --- | --- | --- | --- | --- |
|  | **Total number of patients**  n=30 | **Score**  median  (IQR) | **Total number of patients**  n=25 | | **Score**  median  (IQR) |
| I felt being taken seriously by the medical expert. | 29 | 5.0  (4.0-5.0) | 25 | | 5.0  (5.0-5.0) |
| The medical expert knew about my medical history. | 28 | 4.0  (3.0-4.8) | 25 | | 4.0  (3.0-5.0) |
| The medical expert oriented me about the procedure of the evaluation. | 30 | 4.0  (3.0-5.0) | 25 | | 5.0  (4.0-5.0) |
| The medical expert took my complaints and limitations into account. | 18 | 4.0  (4.0-5.0) | 17 | | 5.0  (5.0-5.0) |
| The medical expert had a pre-conceived opinion about me. | 23 | 1.0  (1.0-3.0) | 23 | | 2.0  (1.0-3.0) |
| Overall perception of fairness (scale from 10 to 0), mean (95%CI) | 30 | 8.0  (7.2 – 8.8) | 25 | | 9.4  (9.1 – 9.7) |
